# Pre-Vaccination Human Papillomavirus Genotypes and HPV16 Variants among Women Aged 25 Years or Less with Cervical Cancer

**DOI:** 10.3390/pathogens12030451

**Published:** 2023-03-13

**Authors:** Yasmin L. Jayasinghe, Sepehr N. Tabrizi, Matthew Stevens, Trishe Y-M. Leong, Jan Pyman, Sonia R. Grover, Suzanne M. Garland, the Early-Onset Cervical Cancer (EOCC) Study Group

**Affiliations:** 1 Department of Obstetrics and Gynaecology, Royal Women’s Hospital, The University of Melbourne, Melbourne, VIC 3010, Australia; 2 Department of Gynaecology, Royal Children’s Hospital, Melbourne, VIC 3010, Australia; 3 Murdoch Children’s Research Institute, Melbourne, VIC 3052, Australia; 4 Women’s Centre for Infectious Diseases, Royal Women’s Hospital, Melbourne, VIC 3010, Australia; 5 The Australian Genome Research Facility, Melbourne, VIC 3050, Australia; 6 Department of Anatomical Pathology, St. Vincents Hospital, Melbourne, VIC 3000, Australia; 7 Department of Anatomical Pathology, Royal Women’s Hospital, Melbourne, VIC 3010, Australia; 8 Department of Paediatrics, University of Melbourne, Melbourne, VIC 3052, Australia

**Keywords:** cervical cancer, young women, human papillomavirus, genotyping, HPV vaccine, HPV16 variants, European variant, Asian American lineage, Asian lineage, E6350

## Abstract

Background: In 2007, Australia introduced a national human papillomavirus (HPV) vaccination program. In 2017, the onset of cervical screening changed from 18 to 25 years of age, utilising human papillomavirus (HPV) nucleic acid testing. The objective of the study is to describe the HPV genotypes and HPV16 variants in biopsies from women ≤ 25 years of age with cervical carcinoma (CC) (cases), compared with those aged >25 years (controls), in a pre-vaccination cohort. Methods: HPV genotyping of archival paraffin blocks (*n* = 96) was performed using the INNO-LiPA HPV Genotyping assay. HPV16-positive samples were analysed for variants by type-specific PCR spanning L1, E2 and E6 regions. Results: HPV16 was the commonest genotype in cases (54.5%, 12/22) and controls (66.7%, 46/69) (*p* = 0.30), followed by HPV18 (36.3%, 8/22 vs. 17.3% 12/69, respectively) (*p* = 0.08). Furthermore, 90% (20/22) of cases and 84.1% (58/69) of controls were positive for HPV16 or 18 (*p* = 0.42); 100% (22/22) of cases and 95.7% (66/69) of controls had at least one genotype targeted by the nonavalent vaccine (*p* = 0.3). The majority of HPV16 variants (87.3%, 48/55) were of European lineage. The proportion of unique nucleotide substitutions was significantly higher in cases (83.3%, 10/12) compared with controls (34.1%, 15/44), (*p* < 0.003, χ^2^, OR 9.7, 95%CI 1.7–97.7). Conclusions: Virological factors may account for the differences in CCs observed in younger compared with older women. All CCs in young women in this study had preventable 9vHPV types, which is important messaging for health provider adherence to new cervical screening guidelines.

## 1. Introduction

In 2017, Australia changed from biennial cytology screening from 18 years of age to five-yearly primary human papillomavirus nucleic acid testing (HPV NAT) commencing from 25 years of age, in line with international recommendations [1]. Just prior to implementation, surveys of Royal Australian and New Zealand college of Obstetricians and Gynaecologist affiliates (*n* = 956), general practitioners and nurse practitioners (*n* = 191) and young women (*n* = 149) demonstrated variable acceptance (50–84%) towards delaying screening to 25 years of age, particularly in women who were unvaccinated, immunosuppressed or had survived childhood sexual abuse [2,3,4]. Two years after the implementation of the revised guidelines, more than 80% of clinicians were comfortable with the extended screening intervals, increased age of first screening and the screening test used [5]. In 2018, the United States (US) Preventive Services Task Force (USPSTF) updated their recommendations for cervical cancer (CC) screening to include the option for primary HPV testing every 5 years for women aged 30–65 years [6]. However, recent reports from the US demonstrate that women aged 21–39 years have a significantly increased chance of being overscreened due to concern about the development of CC [7,8]. An understanding of HPV virology in young women who develop CC prior to the recommended age of onset of screening would be helpful to verify that there are no additional biological factors contributing to more aggressive rapid-onset disease at a young age.

HPV has a circular double-stranded genome consisting of an upstream regulatory region, early genes (E1–E7) and late genes (L1–2). HPV types are classified according to the L1 nucleotide (nt) sequence [9]. Proteins encoded by the early genes are involved in viral persistence, pathogenicity and malignant transformation, whereas the late genes encode the capsid protein [9]. Carcinogenicity of high-risk (HR) compared to low-risk (LR) HPV types is based on nt sequence changes in the early genes, whereas immunogenicity resides in the late region [9]. Epidemiological studies have identified 12 carcinogenic HR-HPV types [10], with types 16 and 18 accounting for 70% of CCs consistently worldwide [11]. HPV16 is unique in terms of being oncogenic, with an odds ratio for the development of squamous cell carcinoma (SCC) of 434.5 [12]. In 1997, Yamada and colleagues reported intratypic HPV16 sequence variations in a sample of 408 cervical cancers from 22 countries and five continents [13]. In this report, the major groupings of HPV16 variants were designated according to 6 major lineages: European (E), Asian (As), Asian American (AA), African 1 (Af1), African 2 (Af2) and North American 1 (NA1). Alphabetical naming is now commonly used for variant lineages/sublineages of HPV, such as A1–3 for the European variants, A4 for Asian lineage, B1–2 for African 1, C for African 2, D1 for North American and D2 for Asian American HPV16 variants [14]. From a public health perspective, it is vital that HPV vaccines provide cross-protection against all HPV16 variants.

In Australia, the national school-based quadrivalent HPV (4vHPV) vaccination program was introduced in 2007 [15]. In December 2014, the U.S. Food and Drug Administration approved the nonavalent HPV (9vHPV) vaccine targeting HR-HPVs 16, 18, 31, 33, 45, 52 and 58, as well as LR-HPVs 6 and 11 [16]. In 2018, a school-based two-dose 9vHPV program spaced 6 months apart was introduced [17]. In 2020, 80.5% of Australian females and 78% of males aged 15 years were reported to have received the full course of the HPV vaccine [18]. Based on the HPV genotypes found in CCs, it is estimated that this vaccine could prevent over 90% of CCs worldwide [19].

There is limited data available on HPV genotype distribution in women aged ≤25 years or HPV variants in CCs of women in Australia. An understanding of this could shed light on cervical cancer biology in the young, inform the predicted impact of HPV vaccines in preventing CC in young women who will not be covered under new screening guidelines and provide important baseline data for the monitoring of vaccine impact in this age group.

In this study, we aimed to describe all HPV genotypes isolated in cervical tissue from women ≤ 25 years of age with CCs, compared with those of older women. In the women positive for HPV16, we aimed to identify HPV variants (in E6, E2 and L1 genes) within the cancer tissue in women ≤ 25 years of age and compare them with those of older women and assess if such changes were silent or resulted in amino acid (aa) changes.

## 2. Materials and Methods

A case-control study was undertaken across gynaecological oncology centres in three Australian states (Victoria, Tasmania, Western Australia): Royal Women’s Hospital (RWH), Mercy Hospital for Women, Monash Medical Centre, Victoria; Royal Hobart Hospital, Tasmania; and King Edward Memorial Hospital, Western Australia. Ethics approval was obtained at all study institutions (respective HREC numbers 06/22; R07/14; 0815/7B; H0010222; 1598/EW).

Participants were diagnosed with CC between 1983 and 2007. Cases were defined as those aged ≤25 years at diagnosis, and controls were aged >25 years at diagnosis. Cases and controls were recruited in a 1:3 ratio. To maximise the number of cases, all subjects who met the case definition were invited as potential participants, while controls were randomly selected using a random number generator [20] and frequency-matched for year of diagnosis (within 5 year intervals). Subjects were identified from medical records databases using International Classification of Diseases codes (Table 1) [21], pathology, oncology databases and state cancer registries for hospital-specific data, where hospital data was incomplete. Four case subjects were recruited from the HPV DNA bank located at the RWH, where purified DNA is stored from fresh CC tissue obtained from 1984 to 1989. The women had given consent for the release of the tissue for research (genotyping of cancer tissue, storage of tissue in the DNA bank and future HPV-related testing). The diagnosis of CC was confirmed histologically.

Women were posted an information sheet and consent form (apart from those who had already consented via the DNA bank). Women who were unable to give consent or who were likely to suffer undue distress were excluded. This included those with language difficulties requiring an interpreter, intellectual disability, recent diagnosis of a terminal disease or unstable psychiatric disorders (psychosis, depression with suicidal ideation). Prior to mail-outs, data was requested from the Australian Electoral Commission (Canberra) and the National Death Index at the Australian Institute of Health and Welfare to minimise the risk of inappropriate mail-outs. A waiver of consent was granted by the ethics committees for the HPV genotyping of tissue of deceased subjects or those lost to follow-up. Chart review was undertaken to collect demographic, survival and histopathological CC data. Socioeconomic indices for area (SEIFA) and decile (range 1–10) were determined by the SEIFA data cubes (2006) from the Australian Bureau of Statistics [22]. The score is derived from census variables, with a lower score indicating an area of relative disadvantage.

Formalin-fixed paraffin blocks of CC tissue were obtained from repositories at the anatomical pathology departments of participating institutions. Seven µm sections were processed for histological analysis by using a sandwich-sectioning method [23]. Detection and genotyping of HPV in CCs were performed at the RWH molecular microbiology laboratory, the WHO Regional (Western Pacific) Labnet for HPV testing. The tissue was deparaffinised according to the manufacturer’s instructions for a Roche DNA Isolation tissue kit (Roche Molecular Systems), as described previously [24]. The INNO-LiPA HPV Genotyping Extra assay (LiPA) version 2 (Innogenetics, Ghent, Belgium), using consensus primers SPF 10 to direct the amplification of a 65-bp region of the HPV L1 gene, was used according to the manufacturer’s recommendations. The assay allows the identification of 28 anogenital HPV genotypes with the inclusion of a 270-bp human DNA (ß globin) internal control and two HPV controls. When multiple HPV infections were present, attribution of the causal agent was made by the a priori risk of cervical cancer and the proportional attribution method according to previous reports [25].

HPV16-positive samples were further analysed for variants by type-specific PCR spanning L1, E2 and E6 regions using primer pairs (Table 2). The 50–100 fmol of purified amplicons were sequenced using 1.6μM of L1, E2 and E6 sequencing primer with a CEQ™ Dye Terminator Cycle Sequencing (DTSC) Quick-Start kit (Beckman Coulter, Inc., Fullerton, CA) according to the manufacturer’s instructions. Both strands of the amplicons were sequenced, and a final contiguous sequence was assembled and aligned using the SeqManProTM. sequence alignment software (Lasergene ^®^, version 5.07, DNASTAR Inc, Madison, WI, USA).

Signature patterns in each gene were used to identify each HPV lineage according to previously published reports [26,27,28] as follows: European (E), Asian (As), African (Af1), African (Af2), North American (NA1) and Asian American (AA). Single nucleotide polymorphisms (SNPs) were defined as follows: (i) the presence of nt changes confirmed by both forward and reverse strands in L1, E2 or E6; (ii) the presence of nt changes in the E6-350 region detected on forward hybridisation alone confirmed the presence of an SNP, as the T-G change at nt 350 and the C-T change at nt 335 are common subclasses [13]; (iii) if substitutions in a gene (apart from the E6-350 region) were detected only in one direction of hybridisation, consistent with a common class or subclass and there were substitutions in other regions of the genome consistent with the same variant, then it was determined highly unlikely that the substitutions arose during PCR alone and the SNPs were included in the analysis; (iv) if substitutions in a gene (apart from the E6-350 region) were detected only in one direction, and there was no supporting data in other regions of the genome, then the result was “indeterminate” for that gene, and that subject was excluded from analysis for that particular gene; and (v) if substitutions in a gene (apart from the E6-350 region) were detected only in one direction but not detected in the other direction, where hybridisation in both directions was successful, the substitutions were presumed to have arisen during PCR and were eliminated, and the subject was included as negative for SNP for that particular gene. Variability in a particular genomic region was defined by the number of unique nt substitutions divided by amplicon length for that genomic region. Nucleotide variability was determined by the total number of nt substitutions divided by the total number of nts examined.

Statistical analyses were performed using STATA IC 11.1 (Statacorp LP, TX, USA). Associations between categorical variables were examined using the chi-square test (χ2) or Fisher’s exact test, and interpreted as odds ratios (OR), 95% confidence intervals (CI) and *p* values (considered significant if ≤0.05). Associations with continuous variables were assessed using the Wilcoxon–Mann–Whitney test. Survival was defined as the number of days from the date of first diagnosis of invasive CC to either the date of death, or for subjects who were alive, the end date of the study, and was reported in years. Five-year survival was defined as the proportion of patients alive at 5 years from diagnosis of CC. Survival rates were compared using Kaplan–Meier curves. Sample size was limited by the number of cases of cervical cancer diagnosed in those ≤25 years of age that could be expected to be recruited over the time of the study.

## 3. Results

### 3.1. Recruitment and Demographic Information

**Figure 1:**
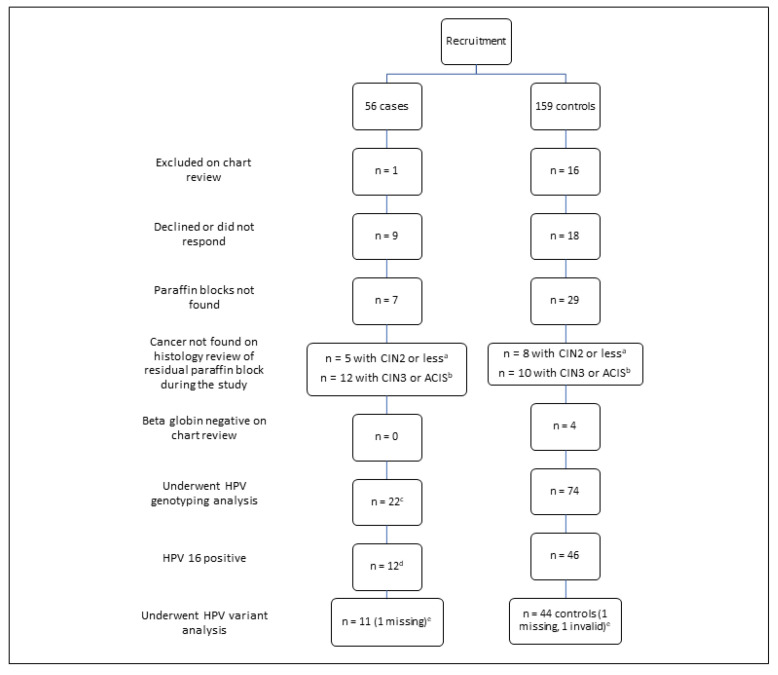
Recruitment and eligibility for HPV testing of cases (≤25 years of age) with cervical cancer and controls (>25 years of age) into the study; ^a^ cervical intraepithelial neoplasia 2 or less; ^b^ cervical intraepithelial neoplasia 3 or less or adenocarcinoma in situ; ^c^ included 4 cases where human papillomavirus DNA purified and banked from fresh cervical cancer tissue (DNA bank); ^d^ included 1 case from HPV DNA bank; ^e^ one case and one control did not have variant testing in time for the study, one control had invalid results on variant analysis (a substitution in E6 (T-145) was seen on reverse hybridisation, but forward hybridisation failed).

Overall, 56 women aged ≤25 years and 159 women aged over 25 years were identified, with 100 women (22 cases, 78 controls) undergoing HPV DNA testing (46.5%). A total of 58 women were HPV16-positive, of whom 56 underwent variant analysis (Figure 1 describes recruitment). There was no significant difference between non-eligible participants (*n* = 115) for mean year of diagnosis (*p* = 0.6), mean SEIFA decile (*p* = 0.6), ethnicity (0.7) or cervical cancer histology (*p* = 0.6) (data not shown). The background characteristics of subjects undergoing HPV detection and genotyping are shown in Table 3. Women aged >25 years with cervical cancer were more likely to be deceased (66.7% vs. 27.3%).

### 3.2. HPV Genotyping Results

The ß globin gene, the internal control used to assess sample adequacy, was positive in all 22 samples from women ≤ 25 years of age and all 22 were HPV DNA-positive. Of the 78 women aged >25 years, 4 (5.1%) were beta-globin-negative, and thus were excluded from further analysis, and 5 were HPV-negative (6.4%). HPV positivity was 94.7% (91/96) in those with valid tests, and all had HR-HPVs.

Overall, 90.8% (20/22) of women aged ≤25 years were positive for HPV16 or 18 compared with 84.0% (58/69) of women aged >25 years (*p* = 0.42, OR 1.9 [0.4–18.9]), whereas 100% (22/22) of women aged ≤25 years had at least one genotype targeted by the 9vHPV vaccine compared with 95.7% (66/69) of women aged >25 years (*p* = 0.3) (Table 4). There was a non-significant trend for HPV18 to be more common in cases (36.3%) than in controls (17.3%) (*p* = 0.06). For the total study population, HPV18 was more common in adenocarcinoma (AC) (6/16, 37.5%) than in squamous cell carcinoma (SCC) (7/63, 11.1%) (*p* = 0.01, 4.8 [1.0–20.5]). However, the proportion with HPV16 was not significantly different between the two morphological types (50.0% (8/16) for AC and 73.0% (46/63) for SCC) (*p* = 0.07, 0.4, [0.1–1.3]). In women aged ≤25 years alone, there was no significant difference in the proportion of SCC (9/12, 75.0%) and AC (6/11, 54.5%) due to HPV16 (*p* = 0.30); however, HPV18 was less common in SCC (1/12, 8.3%) than in AC (3/5, 60%) (*p* = 0.02).

**Figure 2:**
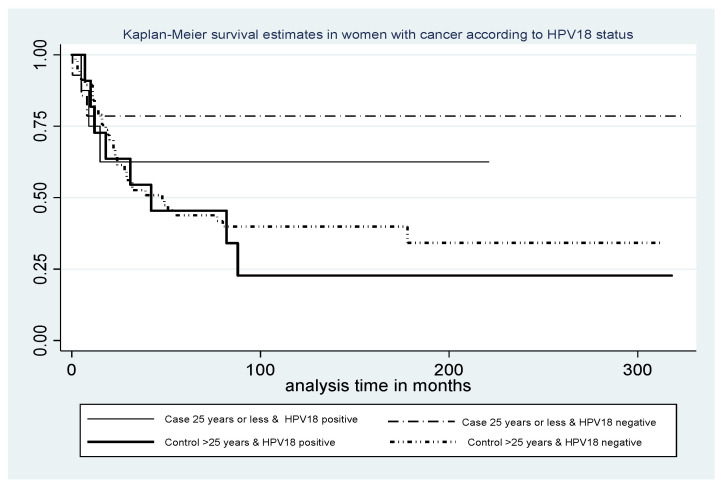
Survival according to HPV18 status in cases (≤25 years of age) with cervical cancer and controls (>25 years of age).

In cases, five-year survival for those who were HPV18-positive was 57.1% (4/7) compared with 78.6% (11/14) for non-HPV18-related cases (Figure 2). For those with Stage I and II disease, five-year survival was 57.1% (4/7) compared with 100% (10/10) for non-HPV18 cancers (*p* = 0.02).

### 3.3. Multiple HPV Infections

The proportion of women with multiple infections was 8.8% (8/91). There was a trend for women aged ≤25 years to have a lower proportion of multiple infections (4.5%, 1/22) compared with women aged >25 years (10.1%, 7/69), but this was not statistically significant (*p* = 0.4, OR 0.4 [0.0–3.6]) (Table 5).

### 3.4. HPV16 Variant Analysis

Fifty-eight HPV16-positive subjects (12 cases, 46 controls) were eligible for variant analysis. Of these, testing was not performed in one case and one control in time for the study. In one control, the variant could not be classified due to an invalid result (a substitution in E6 (T145) was seen on reverse hybridisation, but forward hybridisation failed). Total nt variability was similar in cases (0.3%, 12/4330) and controls (0.3%, 49/17,999) (Table 6). Genomic variability was not significantly different in cases (2.0%, 10/499) compared with controls (3.0%, 15/499) (*p* = 0.3, OR 0.6, 95%CI 0.3–1.6). For all subjects tested, genomic variability was 1.1% (2/169) in L1, 4.5% (5/110) in E2, 5.9% (7/118) in E6 and 3.9% (4/102) in E6 350. The proportion of unique substitutions was significantly higher in cases (83.3%, 10/12) compared with controls (34.1%, 15/44) (*p* < 0.003, χ^2^, OR 9.7, 95%CI 1.7–97.7), which translated to a difference in the proportion of unique aa changes between cases (66.7%, 8/12) and controls (27.3%, 12/44) (*p* = 0.01, OR 5.3, 95%CI 1.1–27.9).

The vast majority of isolates (48/55, 87.3%) were characteristic of the European lineage (Table 7). There was no significant difference in the proportion of European variants compared with non-European variants in cases or controls (*p* = 0.7, χ^2^, OR 1.6, 95%CI 0.6-), nor in the proportion of the T350G variant (3/9, 33.3% compared with 19/38, 50.0%, respectively) (*p* = 0.4, χ^2^, OR 0.5 [0.1–3.8]).

The European variant was the most common variant in both SCC and AC (Table 8). AA variants were seen less commonly in SCC (3/44, 6.8%) compared with other morphological types (3/11, 27.3%) (*p* > 0.05) (Table 8).

## 4. Discussion

There is a paucity of research examining early-onset cancers in young women. This is one of the first studies to examine the genotypes and HPV16 variants in young women to assess if virological factors contribute to a more rapid progression to invasive cancer. In this pre-vaccination study, we found a very high proportion of CCs in women aged ≤25 years attributable to HPV16 and 18 (90.8%), suggesting a predilection of these types for young women. Women aged ≤25 years were found to have restricted HR-HPV genotype distribution (2 types apart from HPV16/18) compared with controls (7 types apart from HPV16/18). Although the total nt and genomic variability were similar between cases and controls, the proportion of unique substitutions was significantly higher in cases (83.5%) compared with controls (34.1%), which translated to a higher proportion of unique aa changes found in cases. Further studies are required to determine if these changes may contribute to differences in viral adaptation, proliferation and, ultimately, early-onset carcinoma.

In a worldwide study of over 10,000 CCs, the proportion of HPV16/18 genotypes found in a subset of 170 CCs from Australia was 79%, with the commonest genotypes being HPV16 (59%), 18 (20%) and 45 (5%), followed by 33, 39 and 53 at 2% each [11]. A study of pre-vaccination CCs in Australia demonstrated that 77.1% (607/787) contained HPV16 or 18, 15.9% (125/787) contained HPV31/33/45/52 or 58 and 7.0% (55/787) contained another HPV type [29]. There was a strong correlation between HPV type and age, with younger women most likely to have HPV16/18 detected, as found in this study.

There is limited data published on HPV variant analysis in young women with CCs. A strength of our analysis was the blinded histology review. Lagstrom et al. found an average of 48.3 variants (range 15–82) per whole HPV16 genome in 15 Dutch women aged 16–29 years; however, the population were women from the community invited for screening [30]. A population study of 160 Argentinian women suggested that the E6 350G SNP was associated with high rates of progression to high-grade cervical disease or CC (OR 19.41 [4.95–76.10]); however, it did not include any women aged ≤25 years with CC [31]. This variant was not more common in our case population (33%) compared with controls (50%), suggesting that it did not play a significant role in causing earlier compared to later onset disease. We found that cases were more likely to have non-synonymous variations (resulting in aa changes) than controls, and further research is required to assess if this may be a mechanism for earlier disease progression. HPV16 lineages were similar in cases and controls (mostly being European variants). This suggests that performing HPV16 lineage analysis on young women with pre-invasive lesions to predict who is more likely to progress to early-disease is of little prognostic value. However, a limitation of the study is the low number of participants with invasive cancer in the case group. Rarer HPV16 polymorphisms could be associated with cancer in younger women and not be revealed by this study. Nevertheless, efforts were made to increase recruitment by including several centres from different regions of Australia to increase the power of the study. There was a trend for HPV16 AA variants to be more common in glandular than in squamous disease, and a larger sample size may have statistically confirmed the association.

We chose variant analysis of HPV16 as it is the dominant genotype in cervical cancer. A limitation of this study is that we did not evaluate the variants of HPV18. There was a trend for HPV18 to be more common in cases with CCs (36.3%) compared with controls (17.3%), suggesting an age-related predilection of HPV18 in cervical neoplastic transformation in younger women. In the absence of other strong biological factors, longer exposure to HPV through unwanted genital contact at a young age may be an important aetiological factor, as it has been shown to independently increase the risk of early-onset cervical disease 5–6-fold [32].

The high proportion of CCs due to HPV16 and 18 (90.8%) suggests that a higher-than-expected proportion of CCs may be prevented in young women with universal vaccination coverage. It is noteworthy that all CCs in young women in this study had preventable 9vHPV types, thus it is likely a very rare outcome in future cohorts, with the potential exception of sexual abuse survivors [32]. Such information is important to relay in targeted education programs to improve adherence to new cervical screening guidelines.

Another limitation of this study is that we found that a significant proportion of CC specimens only had residual high-grade disease left within the paraffin block and had to be excluded (Figure 1). This was more likely in cases due to the high proportion with microinvasive disease and the cancer being sectioned out of the block onto histopathology slides during the original diagnosis. Many of these original reports stated the presence of carcinoma in situ with a small focus of microinvasion of 1–3 mm. Laser capture micro-dissection (LCM) has proven that CCs are clonal (one virus for one lesion) [33]. Microdissection has demonstrated that HPV types in tissue flanking CCs are concordant with genotypes in the lesion [34]. However, the possibility of a separate contiguous preinvasive lesion could not be ruled out, supporting their exclusion from analysis. Multiple infections were found in 8.8% of women in this study, which is similar to other studies which have found multiple infections in 7.7% to 11% of Australian CCs after using strict quality control methods to avoid contamination [29,35]. In our study, adjustment for multiple infections did not make a significant difference to the total proportion of subjects who were HPV16- or 18-positive.

HPV genotype variation over time in CCs is another important factor in estimating the long-term impact of vaccines. We demonstrated that the L1 gene was highly conserved in young women and controls (genomic variability 1.1%), and this together with the knowledge that HPV types have evolved very slowly, and have diverged since the origin of humanity only by about 5% [36], means that we can be comfortable that currently, HPV variants in the Australian population are unlikely to significantly affect vaccine immunogenicity and efficacy in the longer term. Pastrana and colleagues created pseudovirions from the five major phylogenetic branches of HPV16 and found that vaccination with HPV16 114K L1 VLPs generated antibodies against all of the pseudovirion variants. They concluded that HPV16 variants should be regarded as belonging to a single serotype for vaccination purposes [37].

## 5. Conclusions

While HPV genotyping in nationally reported CCs is unknown for this age group, this study provides important baseline data for the monitoring of 4vHPV and 9vHPV and predicting the impact of the revised cervical screening guidelines in those aged <25 years. All CCs in young women in this study had preventable 9vHPV types, which is important messaging for health provider adherence to the new cervical screening guidelines. Accordingly, we advise genotyping surveillance of all CCs diagnosed.

## Figures and Tables

**Table 1:** Diagnosis codes for identification of subjects with primary cervical cancer.

Year	General Diagnosis Code	Specific Diagnosis Code
1983–June 1998	180 Malignant Neoplasm of Cervix Uteri	180.0 Endo Cervix
		180.1 Exocervix
		180.8 Other specified sites of cervix
		180.9 Cervix Uteri, unspecified
July 1998–2007	C53 Malignant Neoplasm of Cervix Uteri	C53.0 Endo Cervix
		C53.1 Exocervix
		C53.8 Overlapping lesion of cervix uteri
		C53.9 Cervix Uteri, unspecified

**Table 2:** Sequence of primers, single nucleotide polymorphism position and length of amplicons generated to identify HPV16 variants.

Gene	Primer Pair	Nucleotide Position Change	Amplicon Length (Base Pairs)
L1	5′-GTTGATACTACACGCAGTAC-3′	6695:6721:6803	169
	5′-ATGTCATAACGTCTGCAGTT-3′		
E2	5′-GCAGTTTGATGGAGACATATGC-3′	3159:3161:3181:3182	110
	5′-CATAATAGTCAACTTGACCCTCT-3′		
E6	5′-TGCAATGTTTCAGGACCCACA-3′	131:132:143:145:178	118
	5′-AGTAACTGTTGCTTGCAGTAC-3′		
E6 T350G	5′-GAATCCATATGCTGTATGTGAT-3′	350	102

**Table 3:** Characteristics of participants with cervical cancer whose samples underwent HPV genotyping.

	Case (≤25 Years) *n* = 22	Controls (>25 Years) *n* = 78	*p* Value, (χ^2^) OR [95%CI]
Deceased status *n* (%)	6 (27.3)	52 (66.7)	<0.001. 0.2 [0.1–0.6]
Median age at diagnosis (years)	24.0	50.5	<0.001(rank-sum)
Interquartile range (IQR)	22–25	40–64	
Total range	18–25	26–92	
Ethnicity *n*%	20 (90.1)	67 (85.9)	0.83
Caucasian	1 (4.5)	6 (7.7)	
Aboriginal, Torres Strait Islander	1 (4.6)	3 (3.9)	
Asian	0 (0.0)	2 (2.6)	
Other			
Median SEIFA decile ^a^	7.5	6.5	
IQR	5–9	4–8	
range	1–10	1–10	
Total	22 (100)	78 (100)	

^a^ Socioeconomic index for area, based on postal/ZIP code. Deciles are based on the postal codes and are not weighted by the population within each postal code. Major city postal codes have, on average, a higher Socio-Economic Indexes for Areas (SEIFA) level and a larger population than regional postal codes. Consequently, the population-weighted median SEIFA decile is about 7, not 5.

**Table 4:** HPV genotypes in cases (≤ 25 years of age) and controls (>25 years of age), adjusted for multiple infections.

HPV Type	Cases *n* (%)	SCC ^a^	AC ^b^	Other ^c^	NE ^d^	Controls *n* (%)	SCC ^a^	AC ^b^	Other ^c^	UD ^e^	Total *n* (%)	*p* Value, OR [95%CI]
16	12 (54.5)	9 (75.0)	2 (40.0)	0	1 (25.0)	46 (66.7)	37 (72.6)	6 (54.6)	2 (40.0)	1 (50.0)	58 (63.7)	0.30, 0.6, [0.2–1.8] ^f^
18	8 (36.3)	1 (8.3)	3 (60.0)	1 (100.0)	3 (75.0)	12 (17.3)	6 (11.8)	3 (27.3)	2 (40.0)	1 (40.0)	20 (22.0)	0.06, 2.7, [0.8–8.9] ^g^
31	0 (0.0)	0	0	0	0	1 (1.4)	1 (32.0)	0	0	0	1 (1.1)	
33	1 (4.5)	1 (8.3)	0	0	0	3 (4.3)	2 (3.9)	0	1 (20.0)	0	4 (4.4)	
39	0 (0.0)	0	0	0	0	2 (2.9)	2 (3.9)	0	0	0	2 (2.2)	
45	1 (4.5)	1 (8.3)	0	0	0	2 (2.9)	1 (2.0)	1 (9.1)	0	0	3 (3.3)	
51	0 (0.0)	0	0	0	0	1 (1.4)	1 (2.0	0	0	0	1 (1.1)	
52	0 (0.0)	0	0	0	0	1 (1.4)	1 (2.0)	0	0	0	1 (1.1)	
58	0 (0.0)	0	0	0	0	1 (1.4)	0	1 (9.1)	0	0	1 (1.1)	
Total	22 (100.0)	12 (54.6)	5 (22.7)	1 (4.6)	4 (18.2)	69 (100.0)	51 (84.1)	11 (15.9)	5 (7.2)	2 (2.0)	91 (100.0)	

^a^ squamous cell carcinoma; ^b^ adenocarcinoma; ^c^ other epithelial cervical cancers (adenosquamous, mucoepidermoid, adenoid basal); ^d^ neuroendocrine cervical cancers; ^e^ undifferentiated cervical cancers; ^f^ HPV16 in women aged ≤25 vs. women aged >25; ^g^ HPV18 in women aged ≤25 vs. women aged >25.

**Table 5:** Cases aged ≤ 25 years and controls aged >25 years with multiple HPV infections.

Subject	Histological Diagnosis	Stage	Specimen	Histology in Block Tested	Year of Diagnosis	HPV Type	HPV Type
Case	mucoepidermoid	IB NOS ^a^	HPV DNA bank	Not applicable	1984	18 ^b^	51
Control	SCC	IB NOS ^a^	Paraffin	cancer	2000	16 ^c^	52
Control	adenosquamous	IIA	Paraffin	cancer	1988	16 ^c^	52
Control	SCC	IBI	Paraffin	cancer	2003	51 ^d^	82
Control	SCC	IIB	Paraffin	cancer	1994	16 ^c^	45
Control	SCC	IAI	Paraffin	cancer	2001	16 ^c^	45
Control	SCC	IIIB	2 Paraffin blocks	cancer	1987	16 ^c^	52
Control	SCC	IIIB	Paraffin	cancer	2005	16 ^c^	18

^a^ not otherwise specified; ^b^ the carcinoma was attributable to HPV18; ^c^ the carcinoma was attributable to HPV16; ^d^ the carcinoma was attributable to HPV51.

**Table 6:** Number of single nucleotide polymorphisms (SNPs) in E6, E2 and L1 in cases aged ≤ 25 years and controls aged >25 years associated with variant lineage and amino acid changes ^a,b^.

HPV Gene	SNPs	Case	Control	Amino Acid Change
L1		*n* = 6	*n* = 23	
	AAc-C6695	1	3	Thr to Pro (T 353P)
	AAc-A6721	1	3	No base change
	subtotal	2	6	
E2		*n* = 10	*n* = 38	
	AA-G3159		2	Thr to Arg (T 135 R)
	AA-A3159	1	2	Thr to Lys (T 135 K)
	AA-T3161	1	2	His to Tyr (H 136 Y)
	AA-C3181		2	Glu to Asp (E 142D)
	AA-A3182	1	4	Ala to Thr (A 143 T)
	subtotal	3	12	
E6		*n* = 10	*n* = 41	
	E-G131		3	Arg to Gly (R 10 G)
	AA-T145	1	5	Gln to His (Q 14 H)
	E-C154	1		No base change
	E-G162	1		Gln to Arg (Q 20R)
	E-A176		1	Asp to Asn (D 25 N)
	As-G178		1	Asp to Glu (D 25 E)
	AA-G183	1		Ile to Arg (L 27 R)
	subtotal	4	10	
E6 350 region		*n* = 9	*n* = 38	
	AA-T335		2	His to Tyr (H 78 Y)
	E-G350	3	14	Leu to Val (L 83V)
	As-G350		1	Leu to Val (L 83V)
	AA-G350		4	Leu to Val (L 83V)
	subtotal	3	21	
Total SNPs	Total SNPs	12	49	

^a^ Of 11 cases and 44 controls, preliminary testing was undertaken in 6 cases and 26 controls for L1, E2 and E6 genes. This revealed little genomic variability in L1 (1.1%), therefore testing for sequence variation in E2 and E6 was undertaken in the remainder of the subjects; ^b^ hybridization or amplification did not occur in all genes resulting in variable denominators.

**Table 7:** HPV16 variant lineages in cases aged ≤25 years and controls aged >25 years.

Variant Class	Case *n* (%)	Control *n* (%)	Total *n* (%)	*p* Value, OR [95%CI]
European	10 (91.9)	38 (86.3)	48 (87.3)	0.7(χ^2^), 1.6, [0.6–79.8]
Asian American	1 (9.1)	5 (11.4)	6 (10.9)	0.8 (χ^2^), 0.8, [0.0–8.3]
Asian	0 (0.0)	1 (2.3)	1 (1.8)	
Total	11 (100.0)	44 (100.0)	55 (100.0)	

**Table 8:** Description of HPV16 variant lineage and cervical cancer morphology.

Morphology	European Variant *n* (%)	Asian American Variant *n* (%)	Asian Variant *n* (%)	Total
SCC ^a^	40 (90.9)	3 (6.8)	1 (2.3)	44
AC ^b^	6 (75.0)	2 (25.0)	0 (0.0)	8
Other epithelial ^c^	1(50.0)	1 (50.0)	0 (0.0)	2
NE ^d^	1(100.0)	0 (0.0)	0 (0.0)	1
Total	48 (87.3)	6 (10.9)	1 (1.8)	55

^a^ squamous cell carcinoma; ^b^ adenocarcinoma; ^c^ other epithelial cervical cancers (adenosquamous, mucoepidermoid, adenoid basal); ^d^ neuroendocrine cervical cancers.

## Data Availability

The data presented in this study are available on request from the corresponding author. The data are not publicly available due to ethical and privacy reasons.
